# Angular Offset Distributions During Fixation Are, More Often Than Not, Multimodal

**DOI:** 10.16910/jemr.14.3.2

**Published:** 2021-06-03

**Authors:** Lee Friedman, Dillon Lohr, Timothy Hanson, Oleg V. Komogortsev

**Affiliations:** Texas State University, San Marcos, Texas, USA; Medtronic Fridley, Minnesota, USA

**Keywords:** Eye movement, eye tracking, fixation, accuracy, multimodality, drift, microsaccades, gaze

## Abstract

Typically, the position error of an eye-tracking device is measured as the distance of the eye-position from the target position in two-dimensional space (angular offset). Accuracy is the mean angular offset. The mean is a highly interpretable measure of central tendency if the underlying error distribution is unimodal and normal. However, in the context of an underlying multimodal distribution, the mean is less interpretable. We will present evidence that the majority of such distributions are multimodal. Only 14.7% of fixation angular offset distributions were unimodal, and of these, only 11.5% were normally distributed. (Of the entire dataset, 1.7% were unimodal and normal.) This multimodality is true even if there is only a single, continuous tracking fixation segment per trial. We present several approaches to measure accuracy in the face of multimodality. We also address the role of fixation drift in partially explaining multimodality.

## Introduction

The spatial accuracy of an eye-tracker is defined as the angular offset between a fixation target and the point of gaze. Accuracy is important for a number of goals, for example: (1) to compare the performance of different eye-eye-trackers (14; 19; 21), (2) to study the visual perception of patients with several eye diseases (8; 26), (3) to assess the neurodevelopment or the development of social skills of infants (12; 20), (4) to study eye-movements during reading (25), and (5) to test a variety of psychological paradigms (23).

According to (14), to calculate the accuracy of an eye-tracker (parentheses added):

''...calibrate your participant and then let him look at a number of points. Record data when the eye is fixating each point and calculate accuracy as the average angular offset (in degrees of visual angle)..." page 168.

Generally, it is assumed that the underlying distributions are unimodal and normal. But if the underlying distributions are multimodal, this measure of accuracy is somewhat less useful. Here are some quotes to support this point of view:

“As a descriptive statistic the mean loses most of its usefulness, for example, since it can be expected to fall between the two modes of a bimodal frequency distribution.'' (6), page 370.

In a section labelled “Multimodal distributions: the mean considered harmful'', we find this quote:

“The mean of a multimodal distribution can lie on an area of low probability, thus being a highly unlikely representative of the distribution.'' (4), page 5.

And:

“The mean of a multimodal distribution, for instance, is not very informative, much less than the modes and their respective weights.'' (11)

We are not aware of any previous research team that has ever statistically tested for multimodality in these distributions or formally tested for a normal distribution. We present evidence that the underlying distributions are, in a considerable majority of cases, not unimodal. Furthermore, when they are unimodal, they are typically not normally distributed. Two previous papers have noticed and discussed the issue of multimodality in fixation stability metrics (5; 26). Both papers offer recommendations for ways to get precision estimates for multimodal fixation distributions. Neither paper provides a solution to the problem of measuring accuracy in the face of multimodality.

In the present study, we will formally test for the multimodality of angular offset distributions in approximately 50,000 distributions from 322 subjects tested twice. Since we do find overwhelming evidence of multimodality, we suggest several metrics for accuracy in the face of multimodality. We compare these various approaches to each other in terms of estimated data quality. For unimodal distributions, we test for Gaussianity, and report the percent of distributions that were normal.

During the review process, the question of the role of drift in accounting for multimodality was raised. According to (14), drift is defined as:

“Accuracy over time: A gradual increasing offset as the recording progresses.” [page 160]

It is not entirely clear whether this term is used in relationship to changes over a task or recording session or is meant to apply to individual fixations. Here, we consider it in the context of individual fixations. It is also not clear to us why very slow movements away from a target should be treated differently from very slow movements toward a target. We operationally define drift as any slow change in angular offset during a fixation, either toward target (lower angular offset) or away from target We relate our measure of drift to our measure of multimodality. If we considered as drift only those slow movements away from target (toward higher angular offset), our results would clearly be different.

## Methods

### The Eye Tracking Database

The eye tracking database employed in this study is fully described in (12) and is labelled "GazeBase". All details regarding the overall design of the study, subject recruitment, tasks and stimuli descriptions, calibration efforts, and eye tracking equipment are presented there. There were 9 temporally distinct "rounds" over a period of 37 months, and round 1 had the largest sample. This report only includes subjects from round 1. Briefly, subjects were initially recruited from the undergraduate student population at Texas State University through email and targeted in-class announcements. A total of 322 subjects (151-F, 171-M) were included. Subjects completed two sessions of recording (median 19 min. apart) for each round of collection. Each session consisted of multiple tasks. The only task employed in the present study was the random saccade task. During the random saccade task, subjects were to follow a white target on a dark screen as the target was displaced at random locations across the display monitor, ranging from ± 15° and ± 9° of visual angle in the horizontal and vertical directions, respectively. The minimum amplitude between adjacent target displacements was 2° of visual angle. At each target location, the target was stationary for 1 sec. There were 100 fixations per task. The target positions were randomized for each recording. The distribution of target locations was chosen to ensure uniform coverage across the display. Monocular (left) eye movements were captured at a 1,000 Hz sampling rate using an EyeLink 1000 eye tracker (SR Research, Ottawa, Ontario, Canada).

### Processing for Each Fixation

Our goal was to study the accuracy for each fixation trial. Although this was not the only possible choice (we could have made a single measurement for each task), analysis at the level of a fixation trial provided the opportunity to evaluate future assessments regarding the role of target eccentricity or pupil size on accuracy or multimodaltiy. With 322 subjects across 2 sessions, and 100 fixations per task, we started with 64,400 fixations. During data processing, described below, the last fixation (#100) was lost. That left 63,756 fixations. Five subjects who had at least one session with fewer than 20 good quality fixations, were excluded. After certain other quality control steps, described below and presented in the results section, approximately 79%, or 50,545 fixations were left.

### Removing Average Saccade Latency

The full gaze position signal contained various eye movements, including fixations, saccades, post-saccadic events and oscillations (PSE), and blinks. We wanted to measure data quality only when subjects were fixating. Typically, the human reaction time to the instantaneous movement of a target (saccade latency) is around 200 ms (18)[p. 113]. We first found the optimal temporal shift of the eye signal for each recording to align the eye and target movements as much as possible. To obtain the best overall estimate of saccade latency, we calculated the mean angular offset distance between the measured gaze position and the target position at shifts of 1 sample, from 1 sample to 800 samples (1 ms to 800 ms). The shift resulting in the lowest mean angular offset distance was chosen. As a result of this shift, fixation #100 was reduced in length and was dropped from the study. The average shift was 237 msec (SD=17, min=192, max=316).

### The Angular Offset Measurement

The error signal we wanted to analyze for accuracy was the angular offset. For each sample, we determine the distance of the horizontal eye position from the horizontal target position and likewise for the vertical signals. Given a position sample (x, y) in degrees of the visual angle, we first converted it to a direction vector using Equation 1:

**Figure:**
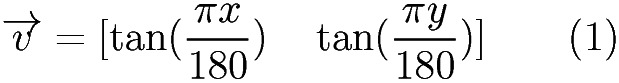


Then, we computed the angular distance between two such direction vectors using Equation 2:

**Figure:**
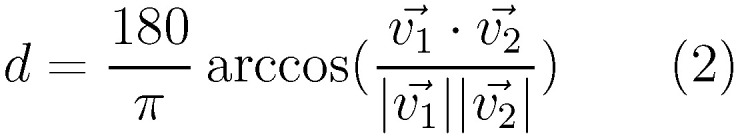


where $\vec{v}$ is the length of some vector in Euclidean space (L2 norm).

It is worth mentioning that for relatively small distances, the angular distance between two direction vectors is very similar to the Euclidean distance between two position samples:

**Figure:**
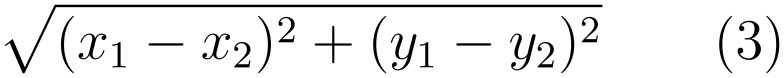


For an angular distance of 10.00^o^, the corresponding Euclidean distance is only 0.5% higher (10.05^o^).

Therefore, the two distance measures are virtually equivalent in practice, and one may prefer the simplicity of Euclidean distance over angular distance.

### Which Part of Fixation to Analyze

**Figure 1:**
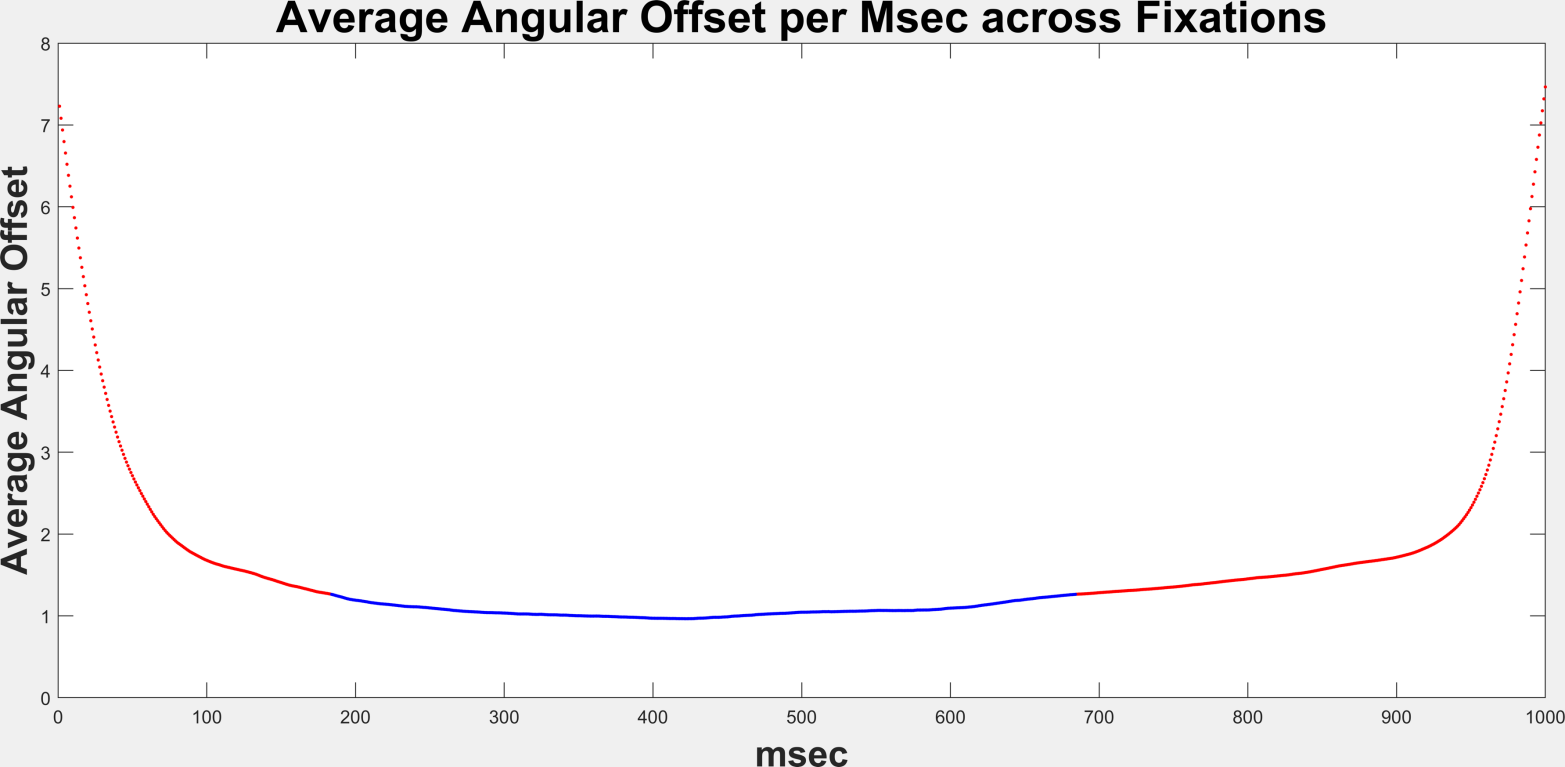
The line represents a plot of the average angular offset per sample. Any fixation which contained any NaN values (likely due to blinks) was not included. Any fixation with any sample with angular offset > 60 deg was also excluded. Of a total of 63,756, only 54,751 fixations were included in these averages. We searched for the stretch with 500 samples with the lowest mean angular offset. This stretch is shown in blue. The rest of the line is red. The low mean stretch started at sample 192 and ended at sampled 691.

We wanted to know which part of the fixation period was least likely to have large error due to saccades. To determine this, we created an average offset per sample, by averaging the angular offset across all studies (N=644) on a per sample basis. Figure 1 below shows the results. The line represents the mean error per sample. The blue colored portion represents the 500 contiguous samples with the lowest mean error. The lowest mean error period started at sample number 192 and ended at sample number 691. This is the data that we ultimately analyzed for accuracy.

The procedures described below for detecting and removing blink saccades and real saccades were largely based on those presented in (22) and (10). In some cases, modifications needed to be made for the present study.

### Removing “Blink saccades”

Blink saccades are pieces of the horizontal and vertical position signals that occur before or after a blink. A blink is indicated by a block of contiguous NaN values in the position traces. Position signals before or after a blink often appear saccade-like and are often confused with saccades. As our goal was to measure the accuracy of fixation, we wanted to remove these blink saccades as well as typical saccades and PSE, and any other part of the signal that did not represent fixation.

Our blink saccade removal method required a threshold on velocity noise during fixation. To compute velocity for each position channel, we used the first derivative of each signal filtered with the Savitzky-Golay procedure (MATLAB, Mathworks, Natick, MA), taking care to perform the analysis without introducing any delay due to the filter. Radial velocity was calculated as the square root of the sum of the squared velocity from both filtered position channels.

The next step was to calculate the 90^th^ percentile of the velocity noise during fixation. Every stretch of signal associated with a peak velocity above 55 deg/sec was removed. This left a series of periods during which fixation might be found (a potential fixation block). If the length of any block was less than 40 msec, the block was rejected. For all remaining blocks, we skipped the first and last 4 samples. For the purposes of this analysis, these sections were treated as fixations. We then created a frequency distribution of the radial velocity of all of the samples in these fixation samples and determined the 90th percentile. A single value was determined for each recording and was referred to as the “fixation velocity threshold'' or “FixVelT''.

To detect the start of a blink saccade, starting at the last good sample before the NaN block, we marched backward in time until three contiguous samples were all below the FixVelT. Of the 3 samples that were all less than FixVelT, the sample closest to the NaN block was taken as the start of the blink saccade (and the end of the prior fixation). To determine the end of the blink saccade we started at the first good sample after the NaN block and marched forward in time until 3 contiguous samples were below FixVelT. Once again, the sample closest to the NaN block was taken as the end of the blink saccade. All of the signal portions related to blink saccades were set to NaN so that they would not be considered in our analysis of the fixations. We visually inspected many of the removed blink saccades and found that this algorithm performed very well.

### Removing Saccades - Step 1

To detect saccades, we found all blocks of data with a radial velocity above 55 deg/sec. These peak blocks were considered to potentially contain the peak velocity of saccades. Each block began at a start sample and ended at an end sample. To find the start of each saccade we marched backward from the start sample until we found a local minimum in the radial velocity that was also less than 30 deg/sec. The end of each saccade was the sample after the end sample of the peak block that was both a local minimum and less than 30 deg/sec. Between the start of each saccade and the end of each saccade, sample values were set to NaN. We visually inspected the performance of the saccade detection and removal procedure and found that it performed well.

### Removing Saccades - Step 2

We found a novel, unusual, and unexpected method of removing other non-fixation events from the recordings. This method was found through trial and error. We performed a regression in a (sliding) window of 27 samples, which started at the 1^st^ sample up to the 27^th^ sample, with the window centered at sample number 14. Regressions were performed in each window, as the window slid from the start of the position data to the end of the position data (last sample number minus window width (27)). The independent variable for each regression was the sample position in each window (1 to 27) raised to the power of 2 (squared). So, the independent variable for the first sample was 1^2^ or 1, and for the last sample was 27^2^ or 789. This regression is similar to a polynomial regression with order 1 (the linear component) removed and only the quadratic component remaining. A statistically significant regression meant that some sort of parabolic function, 27 samples long, fit the data well. The analysis was conducted with the first dependent variable as horizontal position followed by the same analysis based on the vertical position.

Each regression produced an r^2^ which indicated the goodness of fit of the model to the data, and a beta-weight, which was related to the amplitude of the parabolic structure found. For convenience, the beta-weight was multiplied by 1000. We empirically determined that windows with an r^2^ greater than 0.6 and a beta-weight greater than 0.55 typically contained either saccades or pieces of saccades which were not found during the previous saccade removal procedure. Most were very small saccades, or else pieces of saccades, or other saccades which had a somewhat unusual velocity profile. Upon visual inspection, this method was very successful in removing all sorts of non-fixation events from the position data.

### Removal of Anticipatory Saccades

Our task was designed so that each fixation trial was exactly 1 second in duration. In such a predictable situation, subjects often anticipate the target jump and make a saccade prior to the target jump. Such saccades are referred to as “anticipatory saccades'' (AS). These events did occur in our data. The saccade portion of each AS was removed by our saccade removal methods. But after an AS, the fixation level would be far from the target, not due to inaccuracy, but because of the AS. We developed a method to detect these elevated fixation levels due to AS and removed them.

To start, we found, within each fixation, all periods where the eye-position offset (horizontal or vertical) was greater than 2°. Contiguous samples with such offsets were considered potential fixation blocks after AS. If any such block of data was shorter than 100 msec, we rejected it as a post-AS fixation. If any block was longer than 100 msec occurred, at least partially, within our sample range of analysis (192 to 691 samples after the target jump) we considered these post-AS fixations.

The sample number of the start of each of these events was logged. We would expect that the probability of an AS would increase over time. As a check that we were finding AS with our method we created a frequency histogram for the number of AS in each fixation period (from 1-99), we then correlated the frequency in each fixation with the fixation number. We found a highly linear relationship (r^2^=0.85) between the time of onset of an AS and the frequency of AS during each of our 99 fixations. Therefore, it was clear that what we were labelling as AS did tend to occur more frequently with time during the task. We considered that this was consistent with these events being AS.

All the major steps in our preprocessing of fixations are listed in Table 1.

**Table 1.** Steps in the Preprocessing of Fixations

Step 1	Remove saccade latency
Step 2	Remove blink saccades
Step 3	Remove saccades – step 1
Step 4	Remove saccades, etc. - step 2
Step 5	Remove anticipatory saccades

### Evaluation of the Success of These Efforts to Remove Non-Fixation Samples

As a result of our steps to remove non-fixation samples from our fixations we hoped that only fixation samples were represented in the angular offset of these fixations. To check this, we examined 500 randomly chosen fixations. Of these, we rated 408 (82%) as containing only fixation samples, 66 (13%) contained PSEs (typically only 1), 20 (4%) contained microsaccades, 2 contained very slow and/or very noisy saccades, 2 contained a piece of a very slow saccade, 1 contained pieces of a blink saccade and 1 contained RIONEPS (1) noise. We considered that these small and/or brief events would not challenge the statement that the overwhelming number of these fixation samples were indeed fixation only.

### Inclusion Criteria for Fixations

There was a maximum of 500 samples for each fixation. Any fixation that had fewer than 400 samples (i.e., > 100 NaN values after all preprocessing), was excluded from further analysis. Any fixation with more than 4 blocks of contiguous NaNs was also rejected.

### Assessing Unimodality

**Figure 2:**
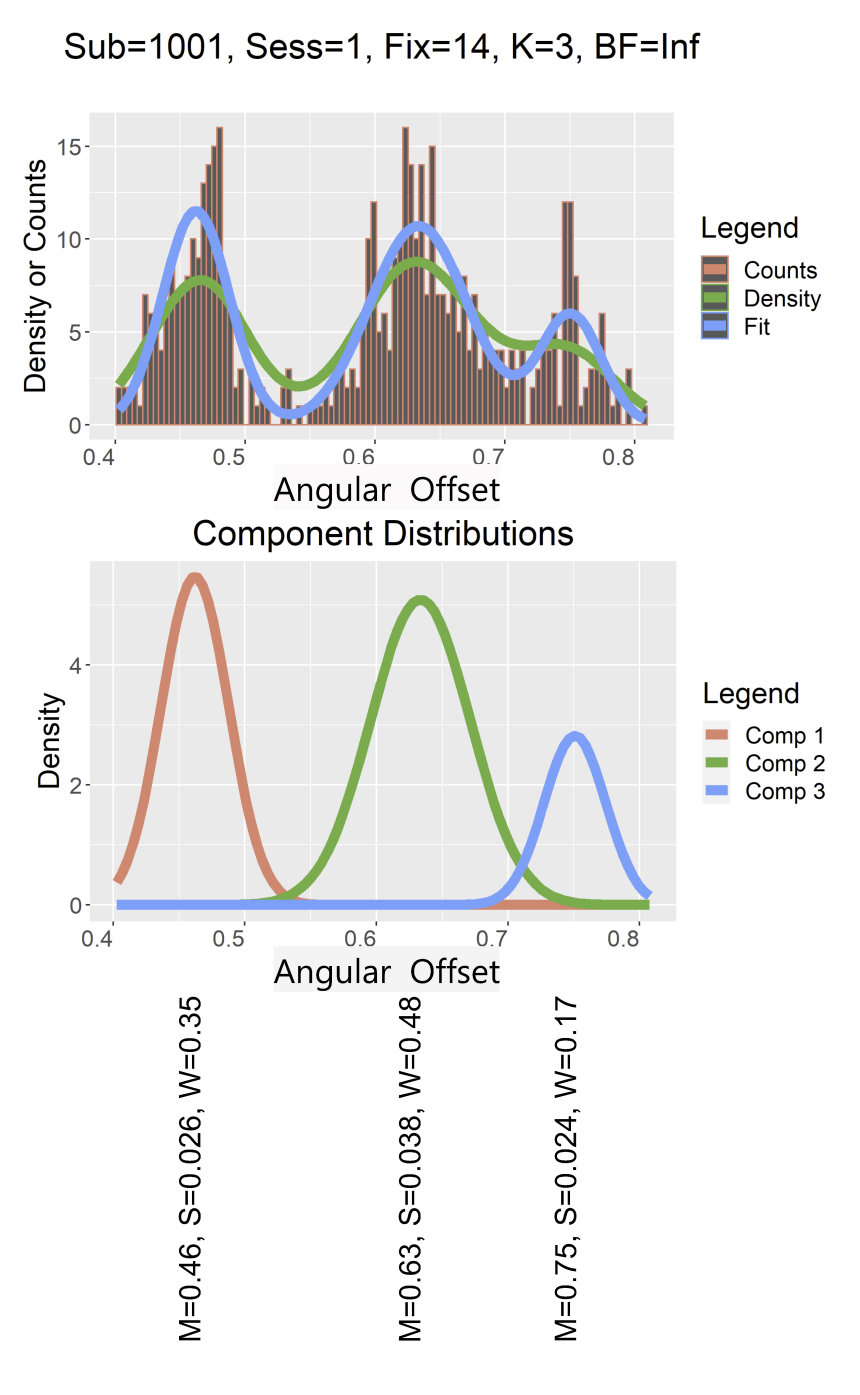
The top panel presents the histogram of angular offsets for subject 1001 (coded number, not consecutive number), session 1, fixation number 14. The green line is the density of the histogram, which can be thought of as a smoothed version of the histogram. The blue line is the fit of the multimodal mixture distribution found by the rjMCMC algorithm. The middle panel displays the three component distributions estimated by the multimodality algorithm. The bottom panel displays the means, SDs and weights of each component estimated by the multimodality algorithm.

To determine if the distributions of angular offset in each fixation were unimodal or multimodal, we employed several methods. First, we employed the Bayesian mixture model approach described in (27) (see Figure 2 for an illustration of this process). The basic idea is that an algorithm is employed to try to fit from 1 to kmax (5, in our case) weighted normal distributions to the histogram of the angular offsets. Each normal component is represented by a mean, a standard deviation (SD) and a weight. The sum of these weights is always 1. This is done repetitively, 2000 times (iterations) and on each iteration, the most likely number (from 1 to 5) of modes in the distribution was determined. The ultimate goal is to determine the Bayes Factor (BF). If a is the prior odds of more than one mode (determined by simulation in our code), and b is the posterior odds of finding more than one mode, then BF=b/a. A log(BF) <=1 means there is no evidence of multimodality (unimodal)(15). A log(BF) between 1 and 3 is considered as positive evidence for multimodality. A log(BF) between 3 and 5 is considered as strong evidence for multimodality. And, finally, a log(BF) > 5 is considered as very strong evidence for multimodality. The algorithm used to perform the mixture model is referred to as a reversible jump Markov chain Monte Carlo (rjMCMC) procedure. The R package that does the fitting is ``mixAK" (16; 17). R code for this computation is available at R code for multimodality testing (24).

In addition, we also applied 1 additional test of multimodality from the ``multimode'' R package (2). Specifically, we employed a version of the excess mass test (ACR). The ACR test is a new multimodality testing procedure that combined 2 approaches, the critical bandwidth approach, and the excess mass approach (ACR).

### Testing for Normality

From a practical point of view, there are problems using classic formal inferential normality testing for large samples sizes. (See Discussion of Formal Normality Testing for a discussion of the issues.) Rather than use these tests, we have come up with our own approach, which is quite similar to the approach of (3), for determining normality. We resample 10,000 pseudo-random normal distributions with the same total N as a plurality of our fixations (500 samples). For each sample normal distribution, we obtain an estimate of skewness and kurtosis. We use these estimates to create 95% confidence limits for skewness and kurtosis. If a test distribution has skewness and kurtosis within these limits, we considered it normal for present purposes. The skewness limits were -0.1821 to 0.1806 and the kurtosis limits were 2.6768 to 3.3655 (the skewness of a normal distribution is 0.0 and the kurtosis of a normal distribution is 3.0).

### Accuracy Metric Names

Accuracy-related fixations consist of all fixations which met the inclusion criteria above (50,545). For each accuracy-related fixation, to estimate accuracy, we calculate the mean (***ClassicAccuracy***). This is the mean of the angular offset distribution regardless of whether the distribution is unimodal or multimodal. We also determine the mean of the component distribution with the maximum weight (***MaxCompMean***). For unimodal distributions we report the median of the fixation-related distributions (***MedianAccuracy***). For unimodal, Gaussian distributions, we report the mean (***MeanAccuracy***).

### Drift

**Figure 3:**
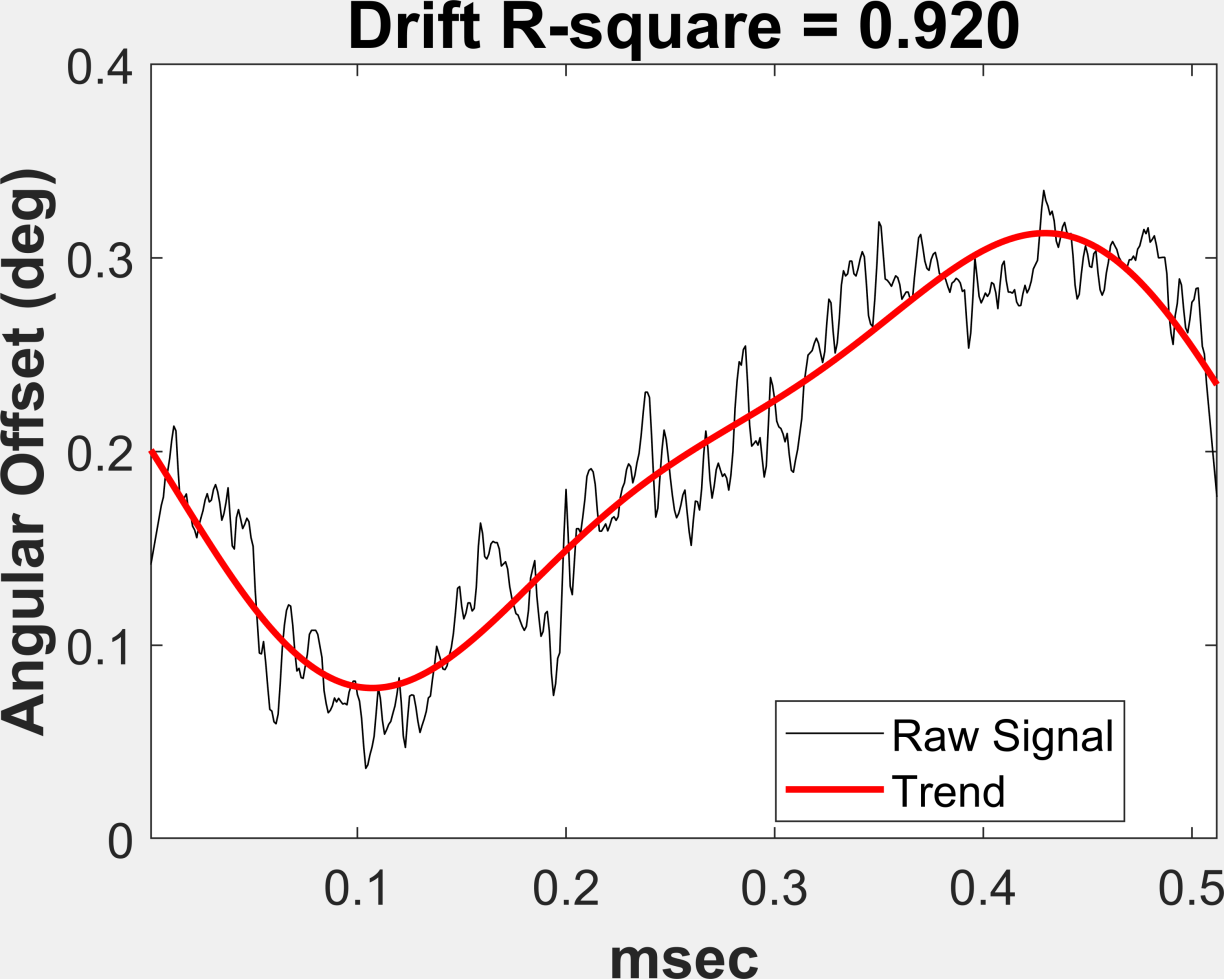
Illustration of a case of high drift.

**Figure 4:**
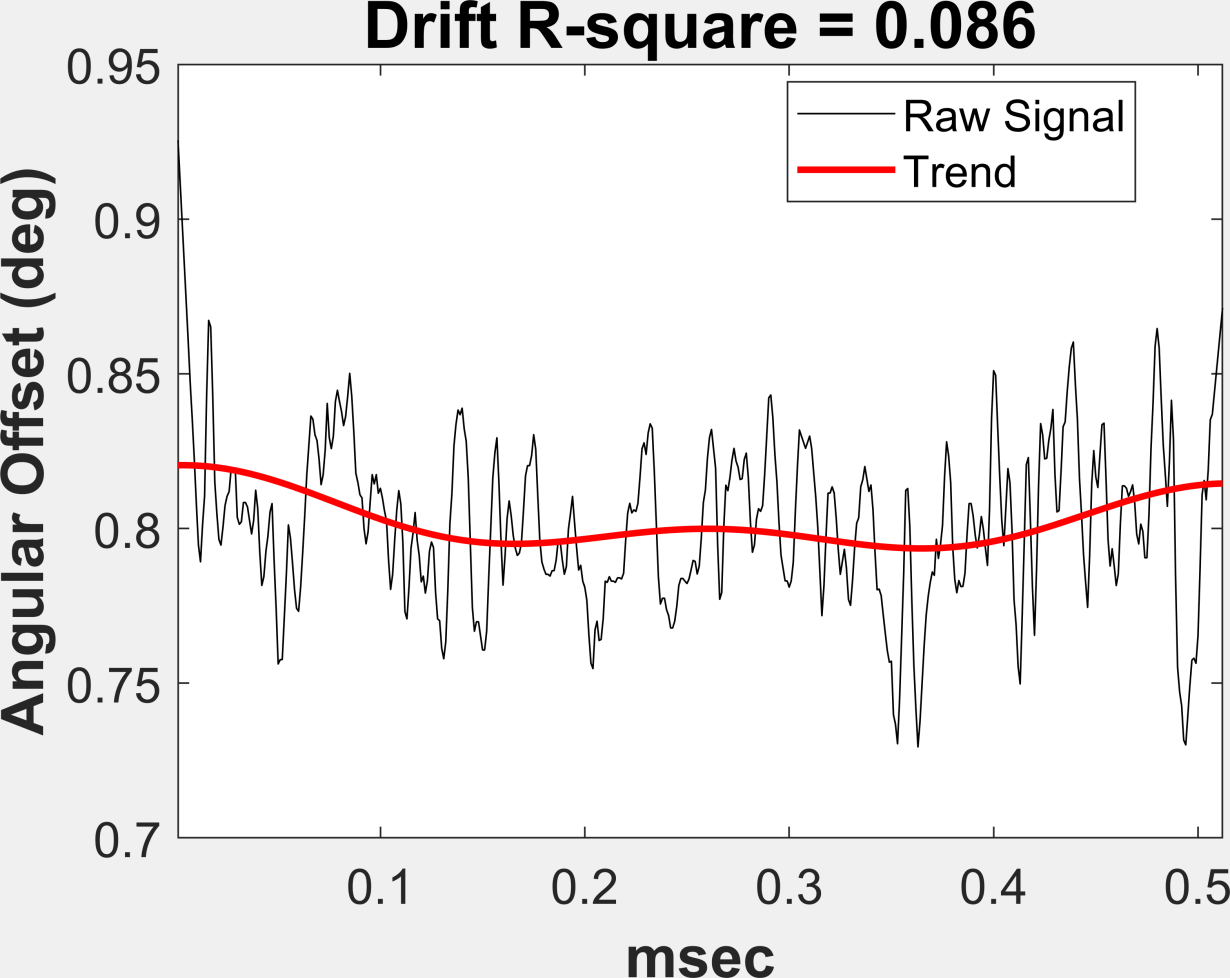
Illustration of a case of low drift

To obtain a measure of drift in the angular offset values, we began by selecting only fixations that consisted of a single, 500 sample fixation segment (N = 14,179). Next, we performed a Fast Fourier Transform (FFT) of the angular offset signal. We retained the slowest frequencies (1.95 Hz and 3.91 Hz and the DC component (mean)) and performed an inverse FFT to obtain a slow frequency curve that matched the angular offset signal. We fit these curves to the angular offset signal by including a linear component and obtained very good fits of the low frequency component to the angular offset signal (see Figures 3 and 4 for examples of high and low drift). The r^2^ of these fits were taken as a measure of drift.

## Results

### Characteristics of Accepted Fixations

**Appendix Figure 1:**
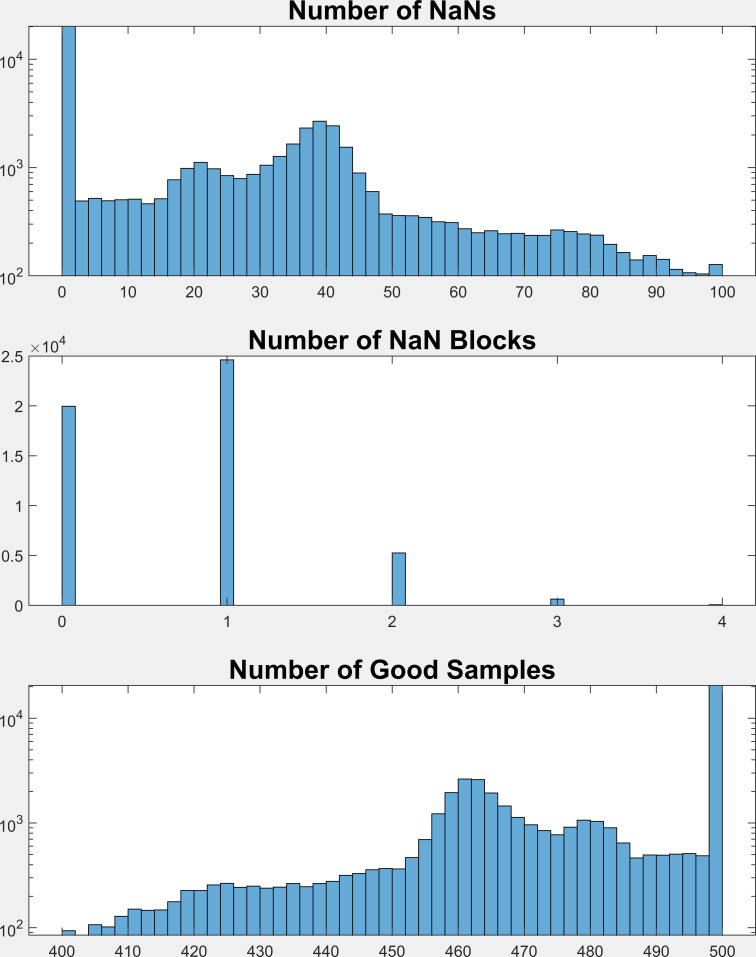
The frequency distributions of Number of NaN values (top), number of NaN Blocks (middle) and number of good data points for fixations (bottom) included in this study

With 322 subjects measured for two sessions, with each session consisting of 99 fixation trials, there was a total of 63,756 fixations to consider. As noted above, to be included in our analysis each fixation had to have at least 400 non-NaN samples, and no more than 4 NaN Blocks (contiguous runs of NaN values, which presumably represent blinks). Also, five subjects with fewer than 20 good fixations for a session were also excluded. This left 50,545 fixations (79% retention) included in our analysis. Appendix Figure 1 illustrates the frequency distributions of number of NaNs, number of NaN Blocks and number of good samples.

### Bayes Factor Distribution

**Figure 5:**
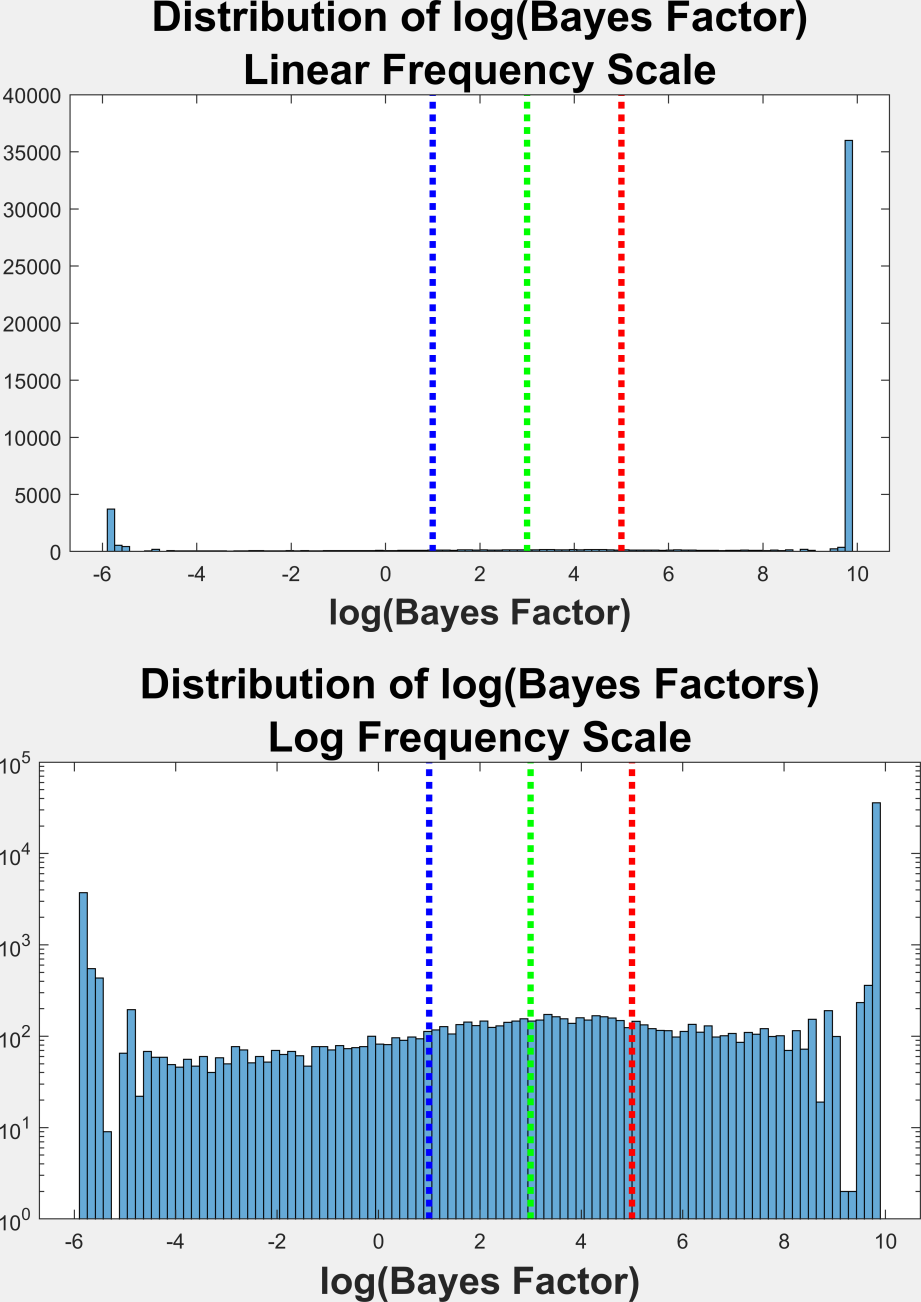
On the top, the frequency distribution of Bayes Factors (log(BF)) across all used fixations for both sessions and all subjects (50,545 fixations.) All BF values that were positive infinite were set to the highest numerical value obtained. All BF values that were negative infinite (log(0)) were set to log(0.003). The lines are the log(BF) thresholds for a value of 1, 3 and 5, corresponding to positive evidence (1 to 3), strong evidence (3 to 5) or very strong evidence (>5) of multimodality. The bottom histogram is the top histogram with the y-scale in log units.

Figure 5 is the frequency histogram of log(Bayes Factors) (logBF) for all fixations in this study. Log(BF) values that were infinite were set to the maximum numerical value found. As noted above, a log(BF) <=1 means there is no evidence of multimodality (unimodal). A log(BF) between 1 and 3 is considered as positive evidence for multimodality. A log(BF) between 3 and 5 is considered as strong evidence for multimodality. And, finally, a log(BF) > 5 is considered as very strong evidence for multimodality. See Table 2 for a breakdown of log(BF) values for fixation trials that contained from 1 to 5 fixation segments. Based on the ACR Test of multimodality, we found 83.5% of all angular offset distributions to be multimodal.

These global values include presented trials which consist of from 1 to 5 fixation tracking segments. Table 2 also presents data for distributions based on from 1 to 5 fixations segments. Although the percentage multimodal is higher for distributions from 2 to 5 segments, even for distributions based on a single, long (400- 500 msec) fixation, the percent multimodal was quite high (69%).

### Histogram of Number of Components

**Figure 6:**
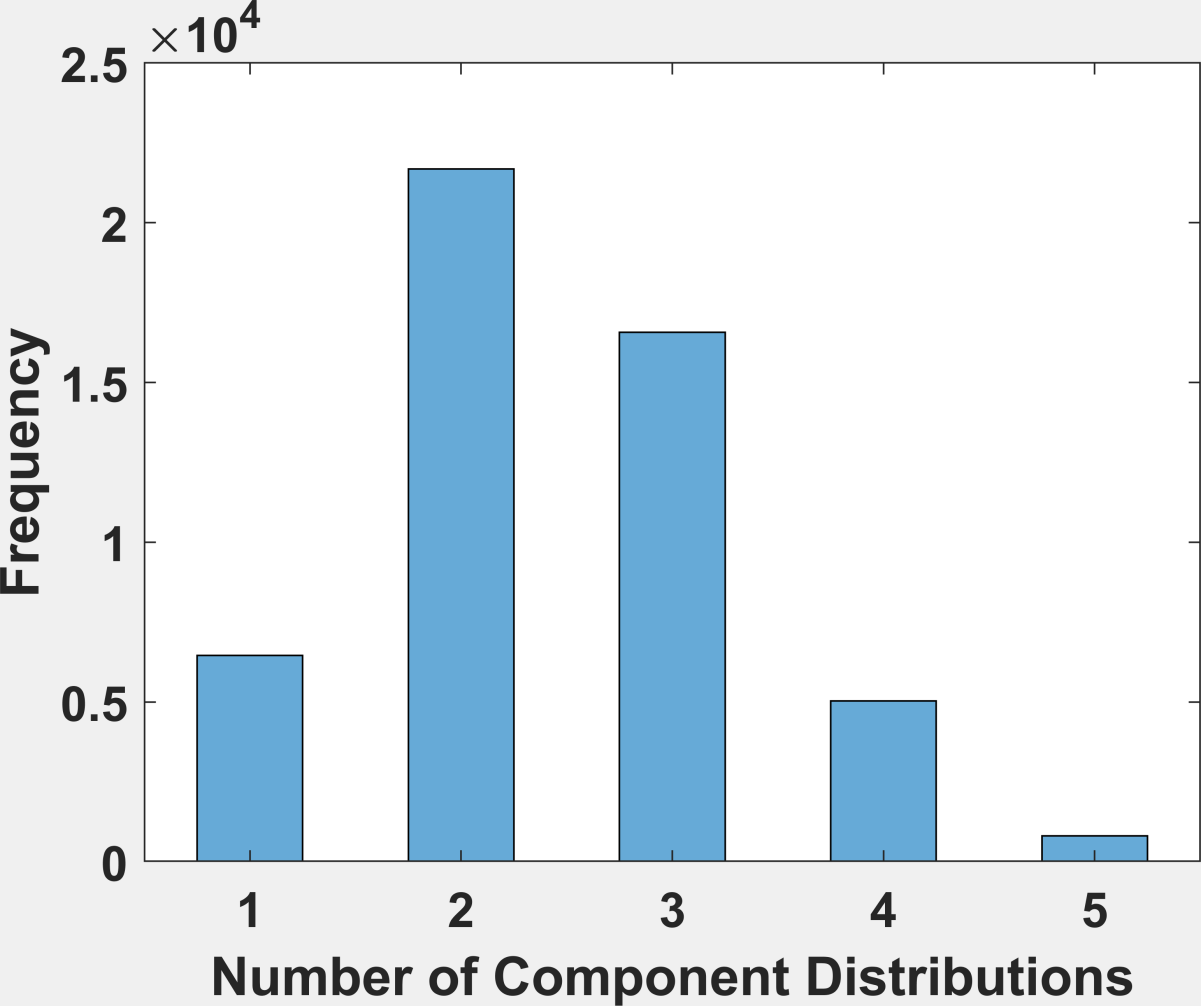
The mixture distribution analysis was allowed to fit from 1 to 5 component distributions. In this figure, we present a frequency histogram of the number of component distributions found. The most frequent number of component distributions is 2.

Figure 6 is the frequency histogram of the number of component distributions found by the multimodality testing algorithm. Two components was the most frequent result and occurred 42.5% of the time. Two or more components were found in 87% of fixations. Evidence of more than 1 component needed to fit a distribution is also evidence of multimodality.

### Distributions of All Measures of Accuracy

**Figure 7:**
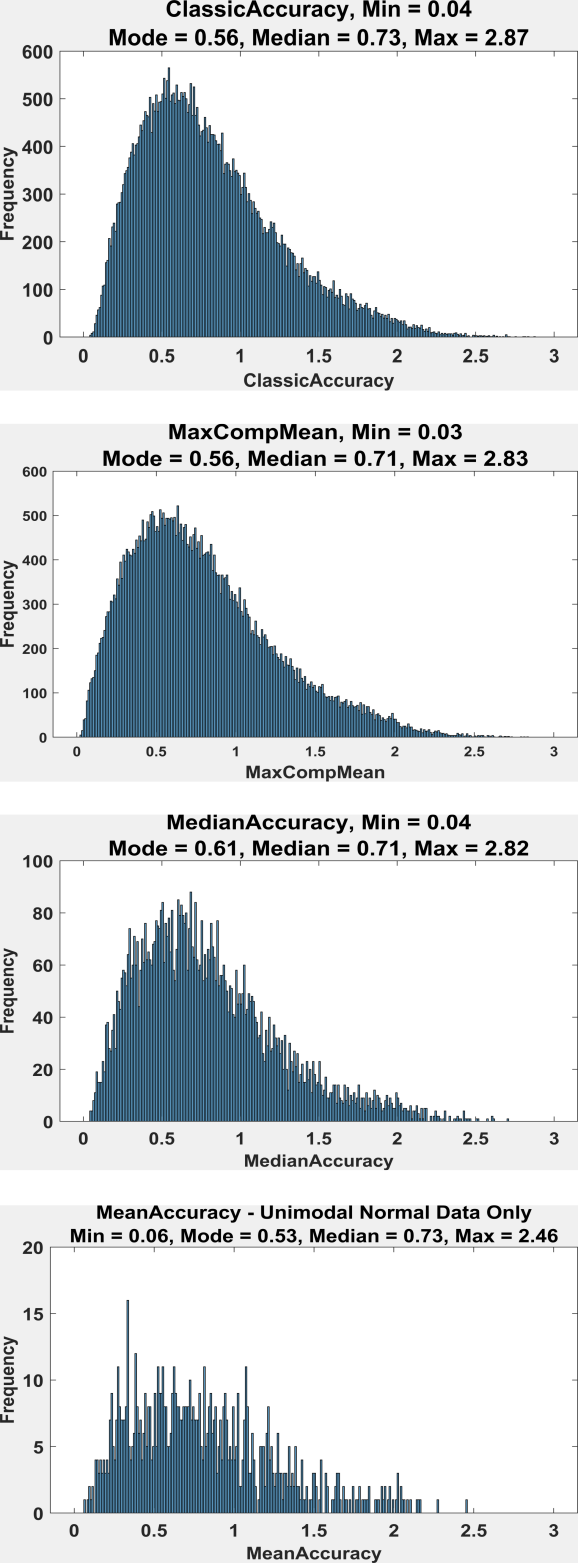
The top is a histogram of ClassicAccuracy measured across 50,545 fixations. The mode in all plots was estimated by the first author. The 2^nd^ from the top is a histogram of MaxCompMean across all fixations. The 2^nd^ from the bottom is the frequency histogram of MedianAccuracy for unimodal distributions only (N=19,461). The bottom is the histogram of the few distributions that were unimodal and met normality criteria (N=855).

Distributions of all four of our measures of accuracy are presented in Figure 7. Note the overall similarity of these distributions, with all having a median between 0.71 and 0.73. This is an indication that our measures that are appropriately applied (all but the top) give more or less the same overall estimate of accuracy as does the ClassicAccuracy (top).

**Table 2.** Percent Multimodal for from 1 to 5 Fixation Segments.

N Fixations	N Events	% Unimodal *	% Positive †	% Strong ‡	% Very Strong $
1	19,961	21.6	4.5	4.9	68.9
2	24,614	11.4	2.8	3.4	82.5
3	5,269	5.47	1.75	2.37	90.4
4	626	6.4	0.3	1.8	91.5
5	75	9.3	2.7	0.0	88.0
Sum	50,545	14.7	3.3	3.9	78.1

*-No evidence of multimodality (log(BF)<=1.) †-Positive evidence of multimodality (log(BF) > 1 & log(BF)<=3.) ‡-Strong evidence of multimodality (log(BF) > 3 & log(BF)<=5.) $-Very strong evidence of multimodality (log(BF) > 5.)

### Relationship Between Multimodality and Drift

**Figure 8:**
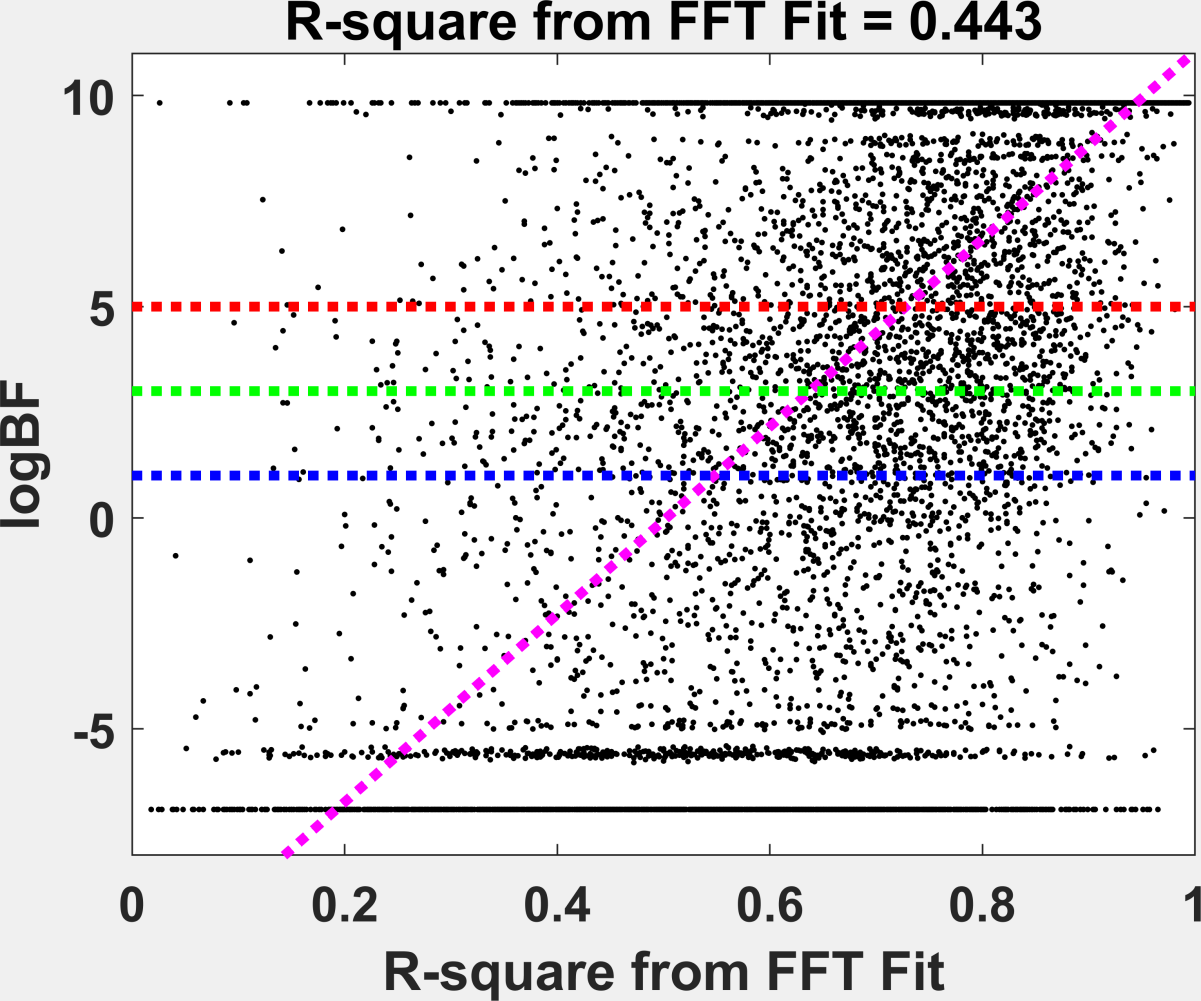
Illustration of the relationship between our measure of drift and our measure of multimodality (log(BF).

**Figure 9:**
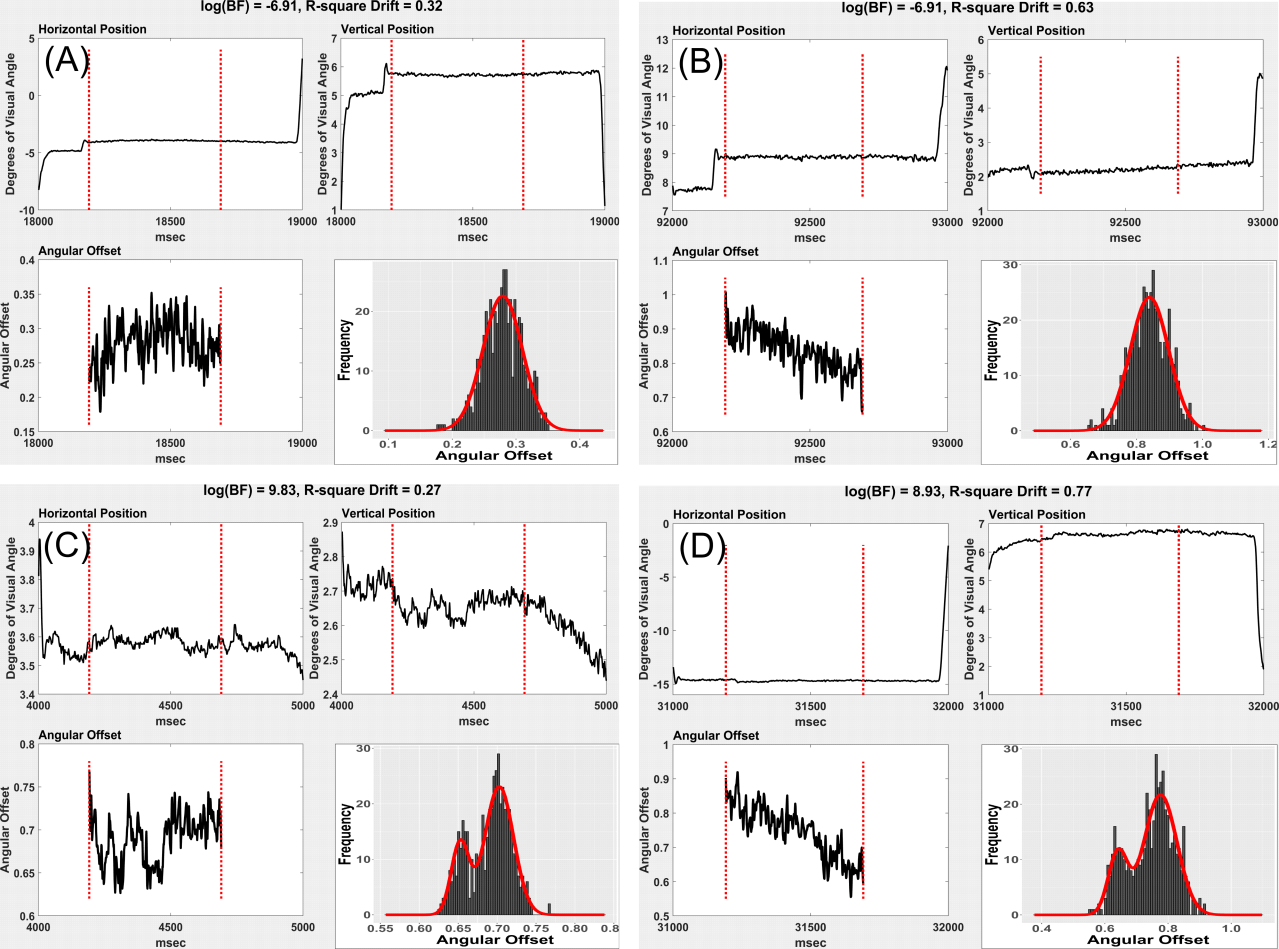
Examples of multimodality and drift. (A) Low drift, low log(BF). (B) High drift, low log(BF). (C) Low drift, high log(BF), (D), High drift, high log(BF). Note that in B and D, drift is toward decreasing angular offset (closer to target).

The relationship between multimodality (log(BF)) and drift is illustrated in Figure 8. The linear relationship between these two measures was highly statistically significant (p < 0.00001) and accounted for 44.3% of the variance. A somewhat dense cloud of points is apparent in the portion of the figure with r2 > 0.55 and log(BF) > 0. It looks like drift during fixation, as we have measured it, does explain a substantial part, but certainly far from all of the variance in multimodality. Below (Figure 9), we provide illustrations of fixations with (A) low log(BF) and low drift; (B) low log(BF) and high drift ; (C) high log(BF) and low drift; and (D) high log(BF) and high drift.. Twelve similar distributions are presented in the Appendix. Note that a number of these drifts are toward decreasing angular offset error.

## Discussion

The main findings of the present study are that distributions of angular offset during fixation are, more often than not, multimodal. In this case, describing the central tendency of these distributions with a mean is not highly useful. We present alternative measures of accuracy that might be more useful. We also report that there is evidence for a relationship between multimodality and a measure of drift, but this relationship leaves much to explain.

The mean of the maximum-weighted Gaussian component found to fit the data (“MaxCompMean”), is interpretable, and is found for every fixation. If one is open to excluding the majority of fixations that are multimodal, the median is appropriate. Only a small subset of these unimodal distributions were normally distributed (1.7% of all fixations). For this small group, the mean is a perfectly fine descriptor of the spread of the distribution (“MeanAccuracy”). The ultimate estimate of accuracy is very similar for each of these measures.

As far as we can tell, there is no generally accepted method for measuring “drift” in individual fixations. We defined drift in fixation as a very slow change in angular offset over time. Although (14) define drift as movement away from target, we considered as drift any gradual change in angular offset during each fixation, either away from or toward the target. Our measure of drift was the model r^2^ of the fit of a slow drift signal to the angular offset data. We did find evidence that drift was positively related to multimodality, and the relationship was substantial. In our view, the relationship was not strong enough to support the view that multimodality is simply a function of drift (compare Figure 10 with Figure 12). Explaining multimodality of angular offsets during fixation is likely to involve a number of factors, which may interact in complex ways. It is the case that sometimes, microsaccades appear to be the basis of multimodality and at other times, microsaccades are clearly not related to multimodality.

### Future Directions

For precision, the differences between each sample angular offset and the angular offset mean for that fixation is determined. The standard deviation of those differences is often taken as a measure of precision. It would be interesting to know if these precision-related distributions were also multimodal. As noted by (13), accuracy is often a function of eye position (eccentricity), a fact that we did not consider for the present analysis. It might be interesting to study if multimodality is also a function of target eccentricity. Similarly, a number of researchers have provided evidence that pupil size affects accuracy (7; 13; 21). Future studies might look for a relationship between multimodality and pupil size.

### Ethics and Conflict of Interest

The author(s) declare(s) that the contents of the article are in agreement with the ethics described in http://biblio.unibe.ch/portale/elibrary/BOP/jemr/ethics.html and that there is no conflict of interest regarding the publication of this paper.

### Acknowledgements

Hal S Stern. Chancellor's Professor, Department of Statistics, UC-Irvine, participated in several discussions regarding the multimodality assessment and we wish to thank him for his important contribution. This work was funded by grant from the NSF (1714623) (PI: Oleg Komogortsev) and NSF Graduate Research Fellowship grant (DGE-1144466) (D. Lohr).

## References

[b1] Abdulin, E., Friedman, L., & Komogortsev, O. V. (2017). Method to Detect Eye Position Noise from Video-Oculography when Detection of Pupil or Corneal Reflection Position Fails, arXiv:1709.02700. Retrieved from https://ui.adsabs.harvard.edu/abs/2017arXiv170902700A

[b2] Ameijeiras-Alonso, J., Crujeiras, R. M., & Rodríguez-Casal, A. (2018). Multimode: An R Package for Mode Assessment, arXiv:1803.00472. Retrieved from https://ui.adsabs.harvard.edu/abs/2018arXiv180300472A

[b3] Cain, M. K., Zhang, Z., & Yuan, K. H. (2017). Univariate and multivariate skewness and kurtosis for measuring nonnormality: Prevalence, influence and estimation. Behavior Research Methods, 49(5), 1716–1735. 10.3758/s13428-016-0814-1 1554-3528 27752968

[b4] Carreira-Perpiñán, M. Á. (2011). Reconstruction of sequential data with density models, arXiv:1109.3248. Retrieved from https://ui.adsabs.harvard.edu/abs/2011arXiv1109.3248C

[b5] Castet, E., & Crossland, M. (2012). Quantifying eye stability during a fixation task: A review of definitions and methods. Seeing and Perceiving, 25(5), 449–469. 10.1163/187847611X620955 1878-4755 22370759

[b6] Clark, M. W. (1976). Some methods for statistical analysis of multimodal distributions and their application to grain-size data. Journal of the International Association for Mathematical Geology, 8(3), 267–282. 10.1007/BF01029273 0020-5958

[b7] Drewes, J., Zhu, W., Hu, Y., & Hu, X. (2014). Smaller is better: Drift in gaze measurements due to pupil dynamics. PLoS One, 9(10), e111197. 10.1371/journal.pone.0111197 1932-6203 25338168 PMC4206464

[b8] Fragiotta, S., Carnevale, C., Cutini, A., Rigoni, E., Grenga, P. L., & Vingolo, E. M. (2018). Factors Influencing Fixation Stability Area: A Comparison of Two Methods of Recording. Optometry and Vision Science, 95(4), 384–390. 10.1097/OPX.0000000000001201 1538-9235 29554006

[b9] Frank, M. C., Vul, E., & Saxe, R. (2012). Measuring the Development of Social Attention Using Free-Viewing. Infancy, 17(4), 355–375. 10.1111/j.1532-7078.2011.00086.x 1532-7078 32693486

[b10] Friedman, L., Rigas, I., Abdulin, E., & Komogortsev, O. V. (2018). A novel evaluation of two related and two independent algorithms for eye movement classification during reading. Behavior Research Methods, 50(4), 1374–1397. 10.3758/s13428-018-1050-7 1554-3528 29766396

[b11] Galtier, N., & Daubin, V. (2008). Dealing with incongruence in phylogenomic analyses. Philosophical Transactions of the Royal Society of London. Series B, Biological Sciences, 363(1512), 4023–4029. 10.1098/rstb.2008.0144 1471-2970 18852109 PMC2607408

[b12] Griffith, H., Lohr, D., Abdulin, E., & Komogortsev, O. (2020). GazeBase: A Large-Scale, Multi-Stimulus, Longitudinal Eye Movement Dataset, arXiv:2009.06171. Retrieved from https://ui.adsabs.harvard.edu/abs/2020arXiv200906171G 10.1038/s41597-021-00959-y PMC8285447 34272404

[b13] Holmqvist, K. (2017). Common predictors of accuracy, precision and data loss in 12 eye-trackers. Unpublished. doi:10.13140/RG.2.2.16805.22246

[b14] Holmqvist, K., Anderssen R. (2017). Eye tracking: A comprehensive guide to methods, paradigms, and measures: CreateSpace Publishing.

[b15] Kass, R. E., & Raftery, A. E. (1995). Bayes Factors. Journal of the American Statistical Association, 90(430), 773–795. 10.1080/01621459.1995.10476572 0162-1459

[b16] Komárek, A. (2009). A new R package for Bayesian estimation of multivariate normal mixtures allowing for selection of the number of components and interval-censored data. Computational Statistics & Data Analysis, 53(12), 3932–3947. 10.1016/j.csda.2009.05.006 0167-9473

[b17] Komárek, A., & Komárková, L. (2014). Capabilities of R Package mixAK for Clustering Based on Multivariate Continuous and Discrete Longitudinal Data. Journal of Statistical Software, 1(12). Advance online publication. 10.18637/jss.v059.i12 1548-7660

[b18] Leigh, R. J., & Zee, D. S. (2015). The Neurology of Eye Movements. Oxford University Press. 10.1093/med/9780199969289.001.0001

[b19] Macinnes, J. J., Iqbal, S., Pearson, J., & Johnson, E. N. (2018). Wearable Eye-tracking for Research: Automated dynamic gaze mapping and accuracy/precision comparisons across devices. bioRxiv, 299925. doi:10.1101/299925

[b20] Morgante, J. D., Zolfaghari, R., & Johnson, S. P. (2012). A Critical Test of Temporal and Spatial Accuracy of the Tobii T60XL Eye Tracker. Infancy, 17(1), 9–32. 10.1111/j.1532-7078.2011.00089.x 1532-7078 32693503

[b21] Nyström, M., Andersson, R., Holmqvist, K., & van de Weijer, J. (2013). The influence of calibration method and eye physiology on eyetracking data quality. Behavior Research Methods, 45(1), 272–288. 10.3758/s13428-012-0247-4 1554-3528 22956394

[b22] Nyström, M., & Holmqvist, K. (2010). An adaptive algorithm for fixation, saccade, and glissade detection in eyetracking data. Behavior Research Methods, 42(1), 188–204. 10.3758/BRM.42.1.188 1554-3528 20160299

[b23] Orquin, J. L., & Holmqvist, K. (2018). Threats to the validity of eye-movement research in psychology. Behavior Research Methods, 50(4), 1645–1656. 10.3758/s13428-017-0998-z 1554-3528 29218588

[b24] R Development Core Team. (2010). R: Language and environment for statistical computing. R Foundation to Statistical Computing.

[b25] Rayner, K., Pollatsek, A., Drieghe, D., Slattery, T. J., & Reichle, E. D. (2007). Tracking the mind during reading via eye movements: Comments on Kliegl, Nuthmann, and Engbert (2006). Journal of Experimental Psychology. General, 136(3), 520–529. 10.1037/0096-3445.136.3.520 0096-3445 17696697

[b26] Whittaker, S. G., Budd, J., & Cummings, R. W. (1988). Eccentric fixation with macular scotoma. Investigative Ophthalmology & Visual Science, 29(2), 268–278.0146-0404 3338884

[b27] Xu, L., Bedrick, E., Hanson, T., & Restrepo, C. (2014). A comparison of statistical tools fori identifying modality in body mass distributions. Journal of Data Science : JDS, 12, 175–196. 10.6339/JDS.201401_12(1).0010 1680-743X

